# Hanafy Ahmed Youssef, DM, MRCS, FRCPsych

**DOI:** 10.1192/bjb.2020.1

**Published:** 2020-04

**Authors:** Omar Youssef

Formerly Consultant Psychiatrist, St Davnet's Hospital, Monaghan, Ireland

**Figure:**
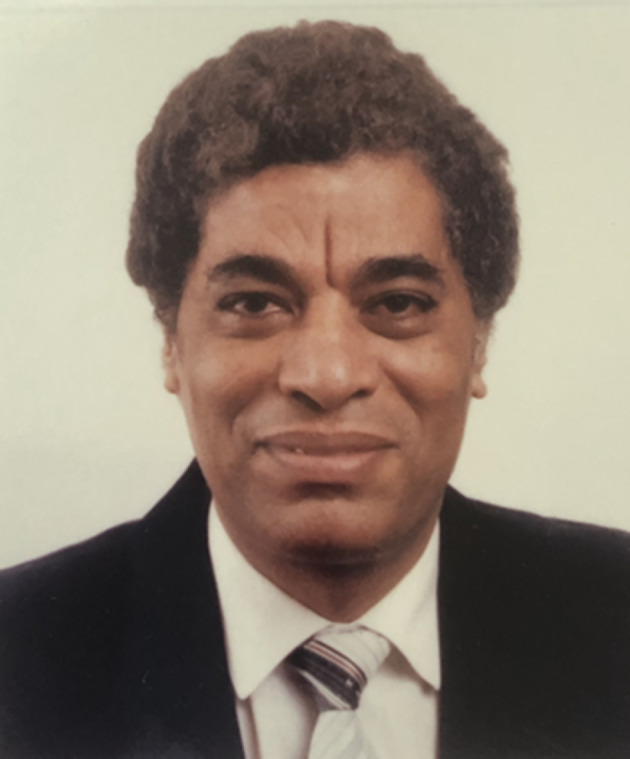

Hanafy Youssef, who died at the age of 80 on 21 January 2019, was a leading figure in Irish psychiatry. With his colleague John Owens, he set up the first community-based psychiatric service in Ireland. He was able to show that this pattern of service led to better outcomes for patients and their families. As a result, a number of other community-based services in Ireland and the UK were established.

Over a 20-year period in the 1980s and 1990s, he developed a highly productive research relationship with Professor John Waddington of the Royal College of Surgeons, Ireland. Together they published many papers on aspects of psychotic illness occurring in patients referred to the Cavan/Monaghan psychiatric services. Hanafy Youssef contributed to over 60 publications in the fields of psychotic illness, neurodevelopmental psychiatry and psychopharmacology. The relationship between the Cavan/Monaghan psychiatric services and the Department of Psychopharmacology at the Royal College of Surgeons, Ireland, continues to this day.

Hanafy had a strong interest in postgraduate psychiatric education. He was clinical tutor at St Davnet's Hospital and a member of the Irish Psychiatric Training Committee. He had a great capacity for explaining difficult concepts to medical and nursing staff. Indeed, as an educator, he was at the forefront of psychiatric education in Ireland. He was also a mentor to many overseas doctors who worked with him. He was an inspiration to them as they saw him become a leader in his specialty. He was a particularly strong supporter of women in medicine at a time when women doctors were still finding it difficult to climb the career ladder.

He had a great interest in the development of psychiatric services in less economically developed countries. In the 1970s he spent time in Zambia, developing clinics in rural areas and helping to confront the stigma often attached to psychiatric illnesses. From 1994, he spent 2 years in a professorial post in Trinidad and Tobago, where he helped improve the quality and prominence of undergraduate psychiatry training. He felt this was crucial if the finest doctors were to be recruited into the specialty. He then returned to take up a consultant post in liaison and general psychiatry at Addenbrooke's, a teaching hospital within Cambridge University Hospitals NHS Foundation Trust. In addition, he carried out voluntary work with charities in Yemen and Libya, providing both general medical and psychiatric care in areas where there was a shortage of doctors.

Hanafy was also a philanthropist, fiercely passionate about social justice, who took seriously the welfare of the less privileged. Together with his siblings he established Latifa's Orphanage House and a mosque in the suburbs of Alexandria. He worked tirelessly for the underprivileged and would offer free medical care to any in need.

Whether they spoke to him in person, heard him speak at international conferences or were his patients, Hanafy Youssef left a lasting impression on all he met. His knowledge, dedication to his field and commitment to his patients were widely appreciated.

He came from humble beginnings. He was born on 14 January 1939 in Alexandria, Egypt, to Ahmed Mahmoud and Latifa (née Taher). His father ran a hunting and tackle store there. Hanafy studied medicine at Alexandria University and undertook postgraduate training in the Department of Psychiatry at the University of Cairo. At university, he led an extremely active life, both in cultural pursuits and in sport. He authored several published novellas in Arabic, won a national poetry award and was the arts reviewer for the university paper. At the same time, he was a member of the university football and wrestling teams. He took an interest in science and politics, and became multilingual, adding French and Russian to the Arabic and English he spoke before entering university. After qualifying as a doctor, he spent 2 years as a medical officer in the Egyptian Army, including service during the Arab–Israeli Six-Day War.

He moved to Derry in 1971 to obtain further training in psychiatry and fell in love with Ireland and its people. This is where he met his wife Ann. He obtained a consultant psychiatrist's post at St Davnet's Hospital, Monaghan, in 1975 and spent most of his career there.

Hanafy retired in 2001 and enjoyed his retirement years in Armagh. He remained highly active, interested in medicine and writing about and closely following the struggle of his Egyptian compatriots for freedom and progress. He published several papers on the history of psychiatry, reviewed books on psychiatry and had several letters published in national broadsheets on a wide range of subjects. He was very proud of his family and their achievements.

He passed away peacefully surrounded by his family. He leaves Ann, his wife of 46 years, five children (Emma, Mahmoud, Latifa, Zahra and Omar) and three grandchildren.

